# Genomic Landscape, Clinical Features and Outcomes of Non-Small Cell Lung Cancer Patients Harboring *BRAF* Alterations of Distinct Functional Classes

**DOI:** 10.3390/cancers14143472

**Published:** 2022-07-17

**Authors:** Alessandro Di Federico, Andrea De Giglio, Francesco Gelsomino, Dario De Biase, Francesca Giunchi, Arianna Palladini, Francesca Sperandi, Barbara Melotti, Andrea Ardizzoni

**Affiliations:** 1 Division of Medical Oncology, IRCCS Azienda Ospedaliero-Universitaria di Bologna, 40138 Bologna, Italy; dr.degiglio@gmail.com (A.D.G.); francesco_gelsomino@aosp.bo.it (F.G.); francesca.sperandi@aosp.bo.it (F.S.); barbara.melotti@aosp.bo.it (B.M.); andrea.ardizzoni2@unibo.it (A.A.); 2 Department of Experimental, Diagnostic and Specialty Medicine (DIMES), University of Bologna, 40126 Bologna, Italy; dario.debiase@unibo.it; 3 Department of Pharmacy and Biotechnology (FaBiT), University of Bologna, 40138 Bologna, Italy; 4 Pathology Department, IRCCS Azienda Ospedaliero-Universitaria di Bologna, 40138 Bologna, Italy; francesca.giunchi@aosp.bo.it; 5 Department of Molecular Oncology, University of Pavia, 27100 Pavia, Italy; arianna.palladini@unipv.it

**Keywords:** non-small cell lung cancer, BRAF, immunotherapy, survival, genomic, non-V600

## Abstract

**Simple Summary:**

Non-small cell lung cancer (NSCLC) patients harboring *BRAF* non-V600 alterations constitute a heterogeneous and poorly studied population orphan of targeted therapies. We conducted a systematic review to detect all *BRAF* alterations of defined functional class across different cancer types. Then, we searched for NSCLC patients harboring these alterations in the cancer bioportal and in POPLAR and OAK trials using patient-level data, to investigate clinical and genomic differences associated with each *BRAF* functional class and the prognostic impact of *BRAF* non-V600 mutations. We found that NSCLC patients harboring distinct classes of *BRAF* alterations have different clinical characteristics, clinical features and genomic landscape. Moreover, *BRAF* non-V600 alterations were associated with a poor prognostic impact, apparently regardless of the treatment received. These peculiar features may suggest the use of tailored treatments according to each class of *BRAF* alteration.

**Abstract:**

Background: In non-small cell lung cancer (NSCLC), BRAF class 1 alterations are effectively targeted by BRAF inhibitors. Conversely, targeted therapies have very low or absent activity in patients carrying class 2 and 3 alterations. The spectrum of BRAF alterations in NSCLC patients, and their accompanying clinical features, genomic landscape and treatment outcomes have been poorly reported. Patients and methods: We identified BRAF alterations of defined functional class across different tumors through a systematic review. Then, we selected NSCLC patients carrying BRAF alterations, according to the systematic review, in the cBioPortal (cBioPortal cohort) to collect and analyze clinical, biomolecular and survival data. Finally, we identified NSCLC patients carrying BRAF non-V600 mutations enrolled in POPLAR and OAK trials (POPLAR/OAK cohort), extracting clinical and survival data for survival analyses. Results: 100 different BRAF non-V600 alterations were identified through the systematic review. In the cBioPortal cohort (*n* = 139), patients harboring class 2 and 3 alterations were more frequently smokers and had higher tumor mutational burden compared to those carrying class 1 alterations. The spectrum of most frequently co-altered genes was significantly different between BRAF alterations classes, including SETD2, STK11, POM121L12, MUC16, KEAP1, TERT, TP53 and other genes. In the POPLAR/OAK cohort, patients carrying non-V600 BRAF alterations were characterized by poor prognosis compared to BRAF wild-type patients. Conclusions: Different classes of BRAF alterations confer distinctive clinical features, biomolecular signature and disease behavior to NSCLC patients. Non-V600 alterations are characterized by poor prognosis, but key gene co-alterations involved in cancer cell survival and immune pathways may suggest their potential sensitivity to tailored treatments.

## 1. Introduction

Non-small cell lung cancer (NSCLC) represents the primary cause of cancer-related deaths worldwide [1]. Recent treatment advances allowed significant extension of the life expectancy of patients diagnosed with locally advanced or metastatic disease. The advent of immunotherapy in non-oncogene addicted NSCLC approximately doubled the median survival, while targeted therapies revolutionized the therapeutic approach for patients carrying actionable oncogenic drivers [2,3,4]. *EGFR* mutations and *ALK* and *ROS1* rearrangements represent the first efficaciously druggable gene alterations in NSCLC [4]. However, more recently, new agents have been found to effectively target other specific molecular alterations such as *BRAF*, *KRAS*, *MET*, *HER2*, *RET* and *NTRK* [5]. Somatic *BRAF* alterations occur in approximately 2–4% of patients with NSCLC, and the V600E mutation has been reported to represent almost half of them [6]. Female sex and smoking history have been described most frequently in NSCLC patients harboring *BRAF* mutations [7,8]. *BRAF* alterations have been classified into three functional classes: class 1 alterations, represented by p.V600X mutations, are characterized by strong activity of BRAF kinase domain and constitutive activation of the MAPK pathway; class 2 alterations, with intermediate to high activity of BRAF kinase domain, activating RAS-independent signaling as dimers; class 3 alterations, characterized by low or complete lack of BRAF kinase domain activity and RAS dependence [9]. Agents targeting BRAF and MEK demonstrated their efficacy in NSCLC patients harboring class 1 *BRAF* mutations, and their use has been recently approved by most regulatory agencies [10,11]. On the contrary, patients whose tumors harbor *BRAF* alterations of class 2 or 3 are currently treated as non-oncogene addicted, since BRAF/MEK inhibitors demonstrated absent or very low activity [12]. However, prevalence, clinical features and treatment outcomes of class 2 and 3 *BRAF* alterations in patients affected by lung cancer are still poorly studied. Previous data suggest that tumors harboring *BRAF* alterations of different classes have distinct clinical characteristics, natural history of disease, and may show different responses to various available treatments. We hypothesized that a distinct molecular landscape might explain those differences, suggesting particular disease features and treatment outcomes. Herein, we first report a systematic review of the literature aiming to identify all *BRAF* gene alterations belonging to a defined functional class across different cancer types. Second, based on the results of the systematic review, we searched for all NSCLC patients harboring *BRAF* alterations of defined functional class in the cBioPortal, with the aim to analyze and compare genomic and clinical features of patients harboring distinct classes of *BRAF* alterations (cBioPortal cohort). Finally, we explored the prognostic impact of BRAF non-V600 mutations on the outcomes of patients enrolled into two randomized controlled trials, the phase II POPLAR trial and the phase III OAK trial (POPLAR/OAK cohort), which demonstrated the superiority of atezolizumab 1200 mg over standard chemotherapy with docetaxel 75 mg/m^2^ in previously treated, squamous or non-squamous, advanced NSCLC patients [13,14,15]. POPLAR and OAK trials were selected for the availability of patient-level and mutation data [15].

## 2. Materials and Methods

### 2.1. Research Strategies

Papers published before 10 June 2021 reporting non-V600 *BRAF* alterations and their corresponding functional class (2 or 3) across all cancer types were searched through the online databases MEDLINE (PubMed) and Cochrane Database of Systematic Reviews and Central Register of Controlled Trials (Wiley, Hoboken, NJ, USA). Records from the Clinical Interpretation of Variants in Cancer (CIViC) were also searched.

Key words used for the research were: “BRAF”; “class”; “type”; “2”; “II”; “3”; “III”; “non-V600”.

Only articles published in peer-reviewed journals and written in the English language were considered.

Studies were retrieved and reviewed by two different authors.

Records underwent a first screening for title and/or abstract. Relevant articles were subsequently screened for full text and analyzed to identify those reporting *BRAF* non-V600 alterations with their respective functional class. Articles reporting non-V600 BRAF mutations that were already listed through previously screened papers or CIViC database were excluded. Articles not reporting the corresponding functional class of the described non-V600 *BRAF* mutation(s) were also excluded. The bibliography of each relevant article was finally searched.

The Preferred Reporting Items for Systematic Review and Meta-Analyses (PRISMA) guidelines were adopted to conduct this work (Supplementary Figure S1).

### 2.2. Study Population

For the cBioPortal cohort, all datasets including NSCLC patients harboring a *BRAF* gene alteration, including mutations, structural variants and copy number alterations (CNA), were searched in the cBioPortal [16,17]. NSCLC patients were subsequently classified based on the functional class of *BRAF* alteration, according to the results of the systematic review, into class 1, class 2, class 3 or unknown functional class. Data about clinical characteristics, genomic landscape, and overall survival (OS) for each patient derived from available online datasets were retrieved from the cBioPortal. For the POPLAR/OAK cohort, patient-level data of participants harboring *BRAF* mutations (all non-V600) were extracted from the available online dataset [15]. Data about clinical characteristics and OS were collected.

### 2.3. Statistical Analysis

Continuous and categorical variables were described as median values and proportions. T-test (or ANOVA or Pearson correlation test or Kruskal–Wallis test if needed) and chi2-test (or Fisher’s exact test, if needed) were performed to compare means and proportions. Shapiro test was performed to verify the normality of data distribution for each variable of interest. A *p*-value ≤ 0.05 was considered statistically significant. The Kaplan–Meier method was used to estimate median survival times. The log-rank test was used to compare survival outcomes. For the POPLAR/OAK cohort, OS was defined as the time from treatment initiation (docetaxel or atezolizumab) to death from any cause [15]. For the cBioPortal cohort, the definition of OS may vary depending on the study analyzed. Top 50 concurrently altered genes in each cohort of NSCLC (BRAF alterations of a known functional class; class 1; class 2; class 3) were retrieved from cBioPortal gene expression data. Mutual exclusiveness of top 50 concurrent gene alterations in each cohort was identified with the Fisher’s exact test, confirmed through the Benjamini–Hochberg false discovery rate (FDR) correction procedure expressed as q-values. Differential expression of top 50 co-altered genes in each class was measured among three BRAF functional classes. Genes differently expressed were identified as those meeting the expression fold-change threshold of absolute value greater than 2 and *p* value ≤ 0.05. *p*-values were adjusted for multiple hypothesis testing via the FDR method. Statistical analyses were performed using RStudio Version 1.3.1093 and the cBioPortal online platform. The following R packages were used: ggplot2; ggrepel; ggstatsplot; DescTools; finalfit; dplyr; knitr; survival; EnanchedVolcano; ggsurvplot; survival.

## 3. Results

### 3.1. Systematic Review

A systematic review of the literature was performed to identify all *BRAF* gene alterations in cancer for which the corresponding functional class was reported.

The initial database search yielded a total of 5977 records. Through reviewing titles and abstracts of each article, 5907 records were excluded as they did not report non-V600 *BRAF* alterations. Full texts of 70 remaining articles were accurately reviewed and analyzed. In total, 53 articles were excluded as they did not report alterations’ functional class or reported already collected variants, in order to avoid duplicates. A total of 17 articles were finally included in the bibliography [9,18,19,20,21,22,23,24,25,26,27,28,29,30,31,32,33]. In total, 27 different non-V600 *BRAF* alterations were listed through the CIViC database [34]. Further, 73 different non-V600 *BRAF* alterations were collected by the 17 selected articles, accounting for a total of 100 different non-V600 *BRAF* alterations listed with the corresponding functional class (Supplementary Table S1).

### 3.2. Study Population

#### 3.2.1. Clinical and Molecular Features and Survival Outcomes

**Figure 1:**
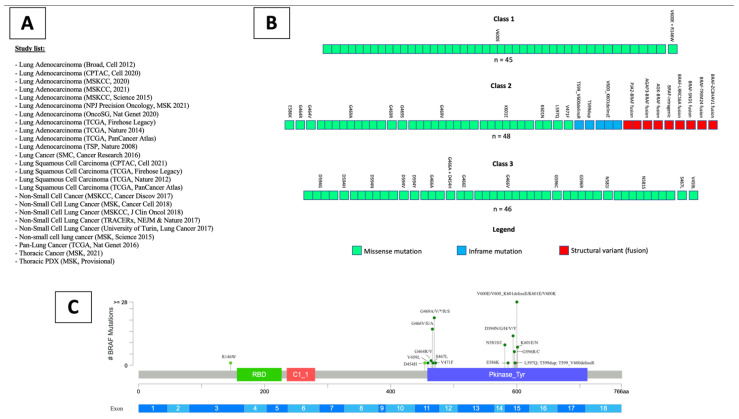
List of studies analyzed through the cBioPortal online platform containing available clinical and genomic data (**A**). Representation of patients (squares) analyzed for each class of BRAF alterations, and corresponding BRAF alteration detected (**B**). Lollipop plot showing the position of detected BRAF class 1, 2 and 3 mutations in the BRAF gene sequence (**C**).

For the cBioPortal cohort, 25 studies including patients with NSCLC were identified in the cBioPortal (Figure 1A) [35,36,37,38,39,40,41,42,43,44,45,46,47,48,49,50]. In total, 4065 patients with a total of 4658 samples were included. Of them, 3132 patients (3553 samples) had adenocarcinoma histology (LUAD), 590 patients (725 samples) had squamous cell histology (LUSC), while the remaining patients had other or not specified histological subtype.

*BRAF* gene alterations were identified in 236/3983 (5.92%) profiled patients with NSCLC. Mutations were found in 198 of 226 (87.6%) patients profiled for mutations, amplifications and deep deletions were found in 31 (14.0%) and 2 (0.9%) of 221 patients profiled for copy number alterations (CNA), and gene fusions were found in 9 of 226 (4.0%) patients profiled for structural variants. In total, 10 NSCLC patients were not profiled for mutations or fusions, and 15 were not profiled for amplifications or deletions. A concurrent *BRAF* mutation and amplification was found in 4 of 221 (1.8%) profiled NSCLC patients. In total, 4 of 226 (1.8%) profiled NSCLC patients had two concurrent different BRAF mutations. No patients with *BRAF* fusion had concurrent *BRAF* amplification or mutation. The prevalence of BRAF alterations was 6.44% (197/3057) in LUAD and 3.6% (21/583) in LUSC. The remaining patients had other or not specified NSCLC histology subtype (Supplementary Figure S2).

According to the results of the systematic review, 45 (1.13%) NSCLC patients (all LUAD) had a *BRAF* class 1 alteration, 48 (1.21%) patients (of whom 45 LUAD) had a class 2 alteration, and 46 (1.15%) patients (of whom 43 LUAD) had a class 3 mutation (Figure 1B,C). The remaining 97 (2.44%) patients had a *BRAF* alteration of unknown functional class, including mutations, splice site variants and CNA (Supplementary Figure S2). Out of the 236 patients harboring *BRAF* alterations, 205 (86.9%) had LUAD histology, 118 (50%) were female and the mean age at diagnosis was 66 (95% CI, 65–68) (Table 1).

**Figure 2:**
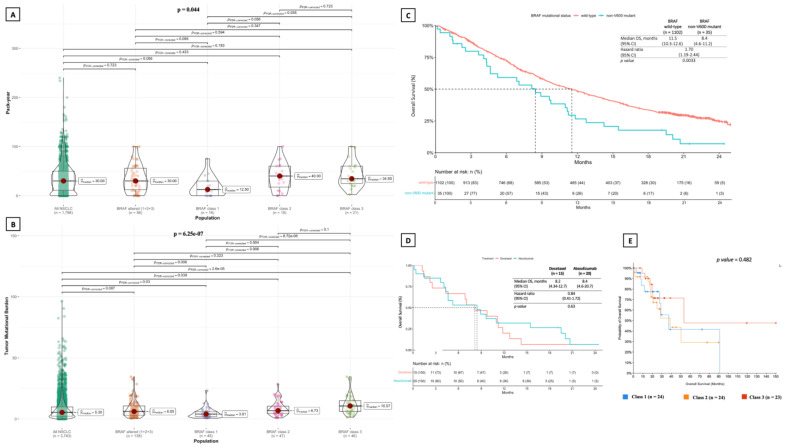
Comparison of smoking habit measured in pack-year among the three BRAF functional classes of alterations, showing a significantly greater pack-year value in patients harboring class 2 and class 3 BRAF alterations as compared to those with class 1 alterations (**A**). Consistently, tumor mutational burden (TMB) was significantly higher in patients harboring class 2 and class 3 BRAF alterations as compared to those with class 1 alterations (**B**). No statistically significant difference in terms of median TMB was found between patients harboring BRAF alterations of known functional class and all patients with NSCLC in the cBioPortal cohort. Kaplan–Meier of overall survival of patients with distinct BRAF alteration classes shows no statistically significant differences between classes in the POPLAR/OAK cohort (**C**), regardless of the treatment type (docetaxel or atezolizumab) (**D**). No statistically significant differences were found in terms of overall survival in patients harboring different classes of BRAF alterations in the cBioPortal cohort (**E**). TMB: tumor mutational burden; NSCLC: non-small cell lung cancer.

According to known smoking habit (175 patients), 146 (83.4%) patients were current or former smokers. Smoking habit was significantly more common among patients harboring class 2 and class 3 alterations than in those with class 1 alterations (*p* for class 1 vs. class 2 or 3 = 0.003), while no difference was documented between patients with class 2 and class 3 alterations (Table 1). Consistently, pack-year was significantly lower in patients with class 1 alterations as compared to those harboring class 3 ones (*p* = 0.035) (Figure 2A). In this cohort (cBioPortal), no statistically significant difference in terms of overall survival (OS) was documented between a total of 71 patients of any disease stage and available survival data harboring class 1 (median OS: 37 months), 2 (median OS: 39 months) and 3 (median OS: 53 months) BRAF alterations (*p* = 0.482; Figure 2E).

For the POPLAR/OAK cohort, 35 patients with previously treated metastatic NSCLC harboring *BRAF* mutations were identified in the POPLAR (*n* = 7) and OAK (*n* = 28) trials (Figure 2C). All of them had *BRAF* non-V600 mutations (12 had class 2 mutations, 10 had class 3 mutations and 13 had *BRAF* mutations of undefined functional class). Patients’ characteristics were consistent with that of the cBioPortal cohort, as they had a mean age of 64 years and almost all of them were previous or current smokers (Table 2).

Out of 35 patients analyzed for survival, 20 (57%) received atezolizumab and 15 (43%) received docetaxel. Patients harboring *BRAF* non-V600 mutations had significantly shorter OS compared to *BRAF* wild-type patients. Median OS was 8.4 (4.6–11.2) months in *BRAF* non-V600 mutated patients versus 11.5 (10.3–12.6) months in BRAF wild-type patients (HR: 1.70; 95% CI, 1.19–2.44; *p* = 0.0033) (Figure 2C). No significant OS differences were observed between *BRAF* non-V600 mutant patients treated with atezolizumab or docetaxel (HR: 0.84; 95% CI, 0.41–1.72; *p* = 0.63) (Figure 2D).

#### 3.2.2. Concurrent Molecular Alterations

**Figure 3:**
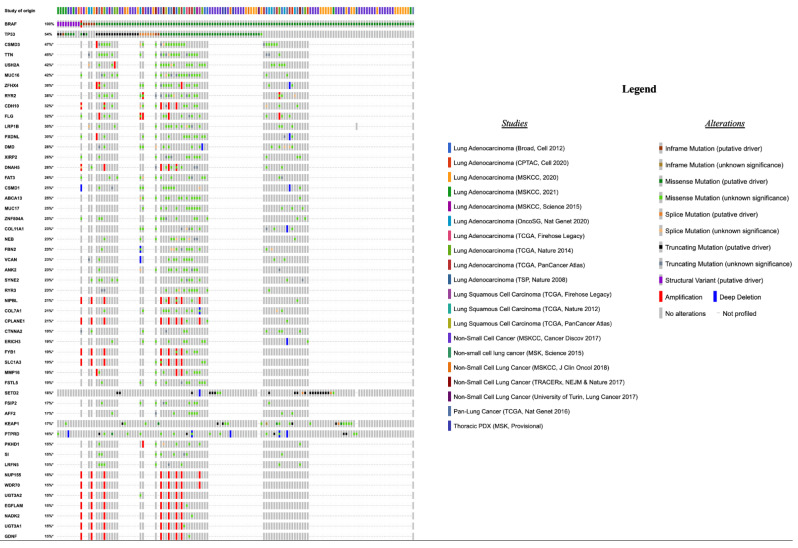
Top 50 most frequently altered genes in patients (*n* = 139) harboring BRAF alterations of any defined functional class, the type of alteration, and the study of origin of each patient.

Among all NSCLC patients with *BRAF* alterations of any of the three functional classes (*n* = 139), *TP53* (75/139, 54%), *CSMD3* (25/53, 47%) and *TTN* (24/53, 45%) represented the most frequently co-altered genes (Figure 3).

**Figure 4:**
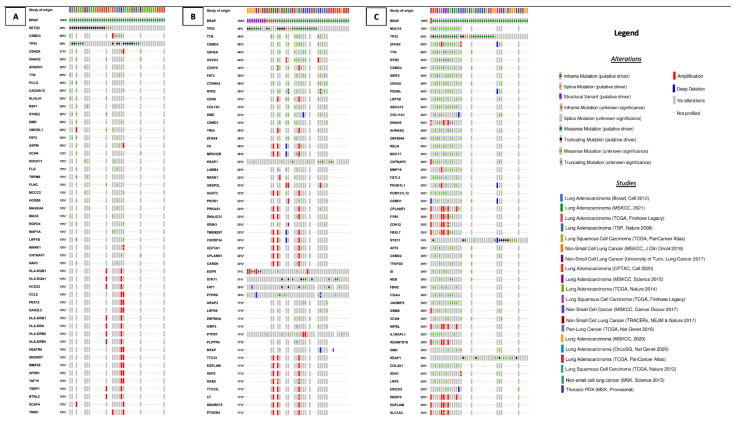
Top 50 most frequently altered genes in patients harboring BRAF alterations of class 1 (**A**), 2 (**B**), and 3 (**C**), the type of alteration, and the study of origin of each patient.

*SETD2* (20/45, 44%), *CSMD3* (6/15, 40%) and *TP53* (17/45, 38%) gene co-alterations were the most common in NSCLC patients with BRAF class 1 alterations (*n* = 45) (Figure 4A), while *TP53* (28/48, 58%), *TTN* (9/18, 50%) and *CSMD3* (9/18, 50%) were the most commonly co-altered genes in patients with *BRAF* class 2 alterations (*n* = 48) (Figure 4B). Finally, among NSCLC patients with *BRAF* class 3 alterations (*n* = 46), the most frequent co-altered genes were *MUC16* (14/20, 70%), *TP53* (30/46, 65%), *ZFHX4* (13/20, 65%) and *TTN* (12/20, 60%) (Figure 4C).

**Figure 5:**
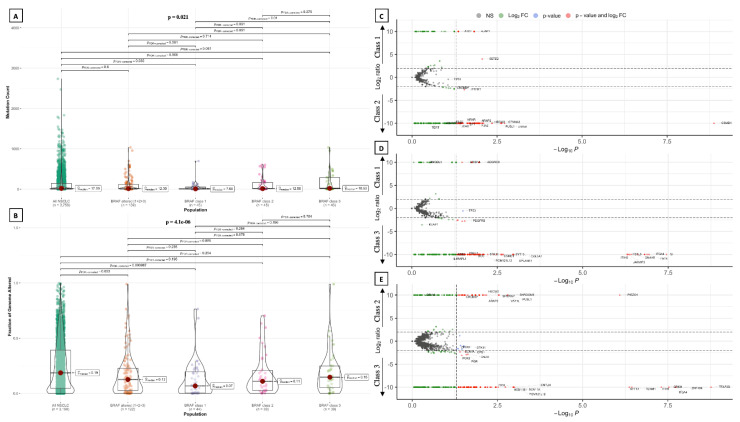
Violin plots show a comparison in terms of mutation count (**A**) and fraction of genome altered (**B**) among the three BRAF functional classes, showing a significantly higher mutation count in patients harboring class 3 alterations compared to those with class 1 alterations. Volcano plots comparing gene co-alteration frequency in patients harboring BRAF alterations of class 1 vs. class 2 (**C**), class 1 vs. class 3 (**D**), and class 2 vs. class 3 (**E**). NS: not significant.

Concurrent gene alterations showed significant heterogeneity among the three BRAF functional classes (Figure 5C–E). In fact, 47 of the most commonly co-altered genes (top 50) and key genes in each class showed statistically significant different co-alteration frequency between the three functional classes of *BRAF* alterations, including *SETD2* (*p* < 0.0001), *STK11* (*p* = 0.0002), *POM121L12* (*p* = 0.001), *MUC16* (*p* = 0.002), *OVCH1* (*p* = 0.003), *ZFHX4* (*p* = 0.004), *ITGA4* (*p* = 0.004), *KEAP1* (*p* = 0.005), *TERT* (*p* = 0.002), *RAS* (*p* = 0.006), *TP53* (*p* = 0.024), *FGFR1/2/3/4* (*p* = 0.042), *ALK* (*p* = 0.047) and DNA damage response and repair (DDR) genes (*p* = 0.049) (Supplementary Table S2). A statistically significant mutual exclusivity in patients with *BRAF* alteration of any class was documented between *STK11* and either *TP53* (*p* = 0.011; q = 0.044) or *TTN* (*p* = 0.004; q = 0.023) co-alterations, and between *PIK3CA* and *XIRP2* co-alterations (*p* = 0.009; q = 0.039).

#### 3.2.3. Tumor Mutational Burden, Mutation Count and Fraction of Genome Altered

Median tumor mutational burden (TMB) was 7.83 mut/Mb (95% CI, 6.85–8.97) in patients with any *BRAF* alteration (*n* = 236). In tumors with *BRAF* alterations of a known functional class (*n* = 139), median TMB did not significantly differ from that of all NSLCL patients (6.05 mut/Mb vs. 5.30 mut/Mb, *p* = 0.209) (Figure 2B). Instead, a statistically significant difference was found between NSCLC harboring distinct classes of *BRAF* alterations (*p* < 0.001). In particular, median TMB was significantly lower in tumors with *BRAF* class 1 alterations (median TMB = 3.91 mut/Mb) than in those harboring class 2 (median TMB = 6.73 mut/Mb, *p* = 0.004) and class 3 (median TMB = 10.57 mut/Mb, *p* < 0.001) alterations, as well as compared to that of NSCLC with *BRAF* alterations of any known functional class (*p* = 0.006) and unselected NSCLC (*p* = 0.03) (Figure 2B). Class 3 alterations were associated with the highest median TMB, even compared to *BRAF* alterations of any known functional class (*p* = 0.006) and unselected NSCLC (*p* < 0.001) (Figure 2B). Total mutation count (MC), defined as the total number of mutations found in each patient’s sample, was not significantly different between NSCLC patients with *BRAF* alterations of a known functional class and unselected NSCLC patients (Figure 5A). However, MC was significantly different among patients carrying distinct *BRAF* alterations classes, and was significantly higher in patients harboring class 3 alterations compared to those with class 1 alterations (*p* = 0.01) (Figure 5A). Likewise, the fraction of genome altered (FGA) was significantly lower in patients with *BRAF* alterations of a known functional class compared to the total of NSCLC patients, but did not show significant differences among the three *BRAF* classes (Figure 5B).

## 4. Discussion

The significance and prevalence of the wide spectrum of *BRAF* gene alterations in cancer is largely unknown. Besides class 1 alterations, encompassing p.V600X mutations, little is known about the role of class 2 and 3 alterations, their prevalence in different tumor types, and their influence on clinical features and treatment outcomes. In NSCLC patients, *BRAF* non-V600 mutations are generally described as half of total *BRAF* mutations [6]. In the current study, following a comprehensive and cross-tumor systematic research of *BRAF* alterations of known functional class, we showed that each class of *BRAF* alterations approximately constitutes 1/3 of total *BRAF*-mutant NSCLC. We also widened the spectrum of *BRAF* alterations by functional classes previously reported in NSCLC patients. Nonetheless, the real prevalence of *BRAF* class 2 and 3 alterations is still to be considered underestimated, as we did not identify any reported corresponding functional class for many alterations found in literature. A deeper knowledge of the significance of these alterations and their clinical implication is thus of paramount importance, as it may lead to a more personalized approach for a considerable number of patients, including the identification of tailored treatments. Uncovering the molecular landscape accompanying *BRAF* alterations of distinct classes constitutes important aid in accomplishing this aim. We showed that class 1 alterations are associated with the lowest median TMB, significantly lower than class 3 ones and unselected NSCLC patients. Conversely, tumors harboring class 3 alterations have a median TMB greater than 10 mutation/Mb, significantly higher than the median of all NSCLC. These results are consistent with a heavier smoking habit in NSCLC patients with *BRAF* class 2 and 3 alterations compared to class 1. The presence of high TMB is a relevant biomarker of high tumor neoantigen load and, by consequence, a possible predictive factor of immunotherapy treatment outcome [51]. However, despite having demonstrated its ability to predict the outcomes of immune-checkpoint inhibitors (ICI) in many studies, the definitive predictive role of TMB in NSCLC is still debated, as its correlation with overall immunotherapy treatment outcome has been inconsistent in terms of survival benefit [51,52,53,54]. A recent retrospective study reported generally unsatisfactory outcomes with immunotherapy in NSCLC patients harboring *BRAF* alterations, although class 2 and 3 altered patients achieved numerically higher objective response rate (ORR) than those carrying class 1 mutations (26% vs. 9%; *p* = 0.25) [55]. Consistently with our study, patients with class 2 and 3 mutations had significantly higher TMB than those harboring class 1 mutations [55]. However, the small sample size, the use of targeted therapy in patients with class 1 alterations and the heterogeneity of lines of ICI treatment constituted important limitations. Results from the IMMUNOTARGET registry, which included 43 NSCLC patients with BRAF alterations, showed higher activity of immunotherapy (ORR: 24.3%) compared to NSCLC patients carrying different oncogene alterations, such as *EGFR*, *MET*, *RET*, *ROS1*, *ALK* and *HER2* ones [56]. Median PFS in BRAF-mutant patients was also longer, especially in those harboring non-V600E mutations, compared to that of patients carrying several different driver gene mutations. However, conversely, median OS was remarkably shorter compared to that of patients carrying other driver alterations, such as *MET* or *RET* ones, supporting the negative prognostic value of *BRAF* mutations [56]. These results are consistent with that of a study from the Israeli Lung Cancer Group suggesting favorable outcomes with ICI in a smaller population of patients with *BRAF*-mutant NSCLC with either V600 or non-V600 alterations, as well as with an analysis of *BRAF*-mutant patients enrolled in the Italian Expanded Access Program of second-line nivolumab [57,58]. Less favorable survival outcomes in *BRAF*-altered patients of class 2 and 3 have also been reported with chemotherapy, mainly due to the presence of more aggressive clinical features compared to NSCLC patients with class 1 alterations, such as a higher frequency of extra-thoracic dissemination [27]. In fact, no survival difference was observed after the exclusion of patients with M1b disease and those treated with targeted therapy [27]. Our results support these findings, suggesting that *BRAF* non-V600 mutations confer a poor prognosis independently of the treatment received. However, a bigger sample size is necessary to determine whether immunotherapy performs better than chemotherapy in this population, and whether patients harboring different classes of *BRAF* alterations derive distinct benefit from specific treatment strategies. In accordance with what was observed with TMB, we showed that the MC and FGA progressively increased from class 1 to class 2 and 3 *BRAF*-altered patients. We also demonstrated that median FGA is significantly lower in patients with *BRAF* alteration of a known functional class compared to unselected NSCLC, but this difference is probably driven by the lower median FGA in patients with class 1 alterations. Similar to TMB, phenotypic implications of MC and FGA may impact patients’ prognosis and immunotherapy efficacy [59,60,61,62,63]. Our work evidenced that distinct classes of *BRAF* alterations in NSCLC are associated with a broad and heterogeneous genomic landscape, and some gene alterations may help in explaining the peculiar behavior of each class. For example, *STK11* and *KEAP1* alterations, which we found with higher prevalence in tumors harboring class 2 and class 3 *BRAF* alterations than in those with class 1 alterations, where they were almost absent, have been associated with high TMB but immune “cold” tumor microenvironment and poor prognosis [64,65,66]. We showed that *TP53* alterations are also particularly enriched in class 2 and, particularly, class 3 *BRAF*-altered NSCLC patients compared to those with class 1 alterations, which may help in explaining the more aggressive behavior of these tumors and the poor outcomes reported in literature [27,67,68,69]. Likewise, we showed that *TERT* mutations, which are rare in lung cancer (approximate prevalence of 2%) and have been correlated with poor prognosis, are enriched in NSCLC patients harboring class 2 and 3 *BRAF* alterations and are absent in V600E mutants, which constituted the totality of class 1 patients [70]. This peculiar distribution among *BRAF* functional classes in NSCLC is in contrast with data from melanoma and thyroid carcinoma patients, where *TERT* mutations have been mainly described in *BRAF* V600E-mutant tumors [71]. We also found *MUC16* alterations in the majority of patients with class 3 *BRAF* alterations, but these occurred very less frequently in patients with class 2 and, especially, class 1 alterations. In melanoma, *MUC16* alterations have been frequently found to be associated with *BRAF* V600E mutations and higher TMB than wild-type patients. Interestingly, these alterations also occur in pancreatic cancer, where they have been associated with disease progression and metastasis through the activation of oncogenic pathways via the interaction between aberrant *MUC16* isoforms and epidermal growth factor (EGF) receptors [72]. On the contrary, consistently with the current literature, *SETD2* co-alterations were present in many *BRAF* class 1 altered patients, but they were infrequent in non-V600 patients [29]. *SETD2* mutations have been associated with high TMB, microsatellite instability and favorable outcomes with ICI [73]. Further gene co-alterations found with high prevalence in one or more *BRAF* functional classes have been associated with higher TMB, such as *TTN*, *CSMD3*, *USH2A* and *RYR2* ones [74,75,76,77]. Together with a different distribution of DNA damage response gene alterations, which has been associated with enhanced ICI efficacy, these features suggest a potentially promising role of immunotherapy in selected patients [78]. The main limitation to our study is represented by the lack of data regarding tumor stage, metastatic sites and treatment outcomes in the cBioPortal cohort, as they were reported for too few patients to allow a proper analysis. Moreover, retrieving data from different studies included in the cBioPortal carries an intrinsic and not avoidable heterogeneity. However, meticulous data screening, cleaning and reporting reduced the risk of misinterpretations. In fact, one of the main strengths of this work is that it is represented by a rigorous methodology, which begins from the detection and collection of BRAF alterations of defined functional class through a comprehensive systematic review of the literature, leading to clinical and molecular data selection, retrieval and analysis from large and high-quality genomic studies and, finally, to the selection of a cohort of patients from two large, practice-changing, randomized clinical trials for survival analyses. Another strength is the production of original data from a large number of patients (271 patients harboring BRAF alterations taking into account both cohorts), considering the rarity of these alterations in NSCLC; moreover, many BRAF alterations of class 2 and 3 have not been previously described and analyzed in patients with NSCLC.

## 5. Conclusions

*BRAF*-altered NSCLCs encompass a broad and heterogeneous genomic spectrum of tumors, each with distinctive molecular signatures, clinical-biological behavior and potentially exploitable specific treatment strategies. NSCLC patients harboring non-V600 *BRAF* alterations constitute a considerable and underestimated population characterized by peculiar genomic landscape and poor prognosis compared to BRAF wild-type patients, warranting larger and deeper studies aiming to identify potential tailored therapies.

## Figures and Tables

**Table 1:** Main clinical characteristics of patients harboring BRAF alterations detected through the analysis of the cBioPortal.

Clinical Characteristics	Total (*n* = 236)	Class 1 (*n* = 45)	Class 2 (*n* = 48)	Class 3 (*n* = 46)	Undefined Class (*n* = 97)	*p* Value
**Age, Mean (SD)**	66.0 (9.4)	66.6 (10.6)	68.5 (9.6)	65.7 (8.8)	64.8 (8.9)	0.243
**Sex, *n* (%)**						
**Female**	118 (51.5)	30 (68.2)	22 (47.8)	27 (58.7)	39 (41.9)	0.023
**Male**	111 (48.5)	14 (31.8)	24 (52.2)	19 (41.3)	54 (58.1)	
**Histology, *n* (%)**						
**Adenocarcinoma**	205 (86.9)	45 (100.0)	43 (89.6)	41 (89.1)	76 (78.4)	0.005
**Squamous**	23 (9.7)	0 (0)	2 (4.2)	3 (6.5)	18 (18.6)	
**Non-Small Cell Lung Cancer NOS**	8 (3.4)	0 (0)	3 (6.2)	2 (4.3)	3 (3.1)	
**Geographical origin, *n* (%)**						
**Caucasian**	65 (73.9)	5 (45.5)	13 (65.0)	12 (100.0)	35 (77.8)	0.059
**Asian**	17 (19.3)	5 (45.5)	6 (30.0)	0 (0)	6 (13.3)	
**African**	6 (6.8)	1 (9.1)	1 (5.0)	0 (0)	4 (8.9)	
**Smoking habit, *n* (%)**						
**Yes**	146 (83.4)	21 (56.8)	31 (93.9)	36 (92.3)	58 (87.9)	<0.001
**No**	29 (16.6)	16 (43.2)	2 (6.1)	3 (7.7)	8 (12.1)	

NOS: not otherwise specified.

**Table 2:** Main clinical characteristics of patients harboring BRAF mutations in POPLAR/OAK cohort.

Clinical Characteristics	Total (*n* = 35)	Class 2 (*n* = 12)	Class 3 (*n* = 10)	Undefined Class (*n* = 13)	*p* Value
**Age, Mean (SD)**	64.1 (9.2)	65.1 (7.5)	65.4 (5.7)	62.1 (12.5)	0.415
**Sex, *n* (%)**					
Female	14 (40.0)	6 (50.0)	3 (30.0)	5 (38.5)	0.581
Male	21 (60.0)	6 (50.0)	7 (70.0)	8 (61.5)	
**Histology, *n* (%)**					
Adenocarcinoma	26 (74.3)	10 (83.3)	9 (90.0)	7 (53.8)	0.091
Squamous	9 (25.7)	2 (16.7)	1 (10.0)	6 (46.2)	
**Geographical origin, *n* (%)**					
Caucasian	27 (77.1)	9 (75.0)	7 (70.0)	11 (84.6)	0.669
Asian	6 (17.1)	2 (16.7)	2 (20.0)	2 (15.4)	
Other	2 (5.7)	1 (8.3)	1 (10.0)	0 (0)	
**Smoking habit, *n* (%)**					
Yes	33 (94.3)	12 (100)	9 (90.0)	12 (92.3)	0.431
No	2 (5.7)	0 (0)	1 (10.0)	1 (7.7)	

## Data Availability

Patient-level data from the POPLAR and OAK trials are available as supplementary information of the Gandara DR et al. [15]. Data extracted from the 25 studies (Figure 1A) included in the cBioPortal are available at www.cbioportal.org, last accessed on 18 February 2022.

## Supplementary Materials

The following supporting information can be downloaded at: https://www.mdpi.com/article/10.3390/cancers14143472/s1, Figure S1: Preferred Reported Items for Systematic Reviews and Meta-Analysis (PRISMA) flowchart of literature research adopted to conduct the systematic review; Figure S2: Consort Diagram summarizing the selection of NSCLC patients harboring BRAF alterations of defined functional class in the cBioPortal; Table S1: List of BRAF alterations (protein change) and corresponding functional class detected through the systematic review of the literature; Table S2: Comparative frequency of concurrent gene alterations in most commonly altered and key genes among NSCLC patients harboring BRAF class 1, class 2, and class 3 alterations; Table S3: Descriptions of detected BRAF structural variants in cBioPortal.
