# Supplementary material for: Genomic Landscape, Clinical Features and Outcomes of Non-Small Cell Lung Cancer Patients Harboring BRAF Alterations of Distinct Functional Classes
Source: Cancers (Basel). 2022 Jul 17;14(14):3472. doi: 10.3390/cancers14143472 (PMC9319412; doi:10.3390/cancers14143472)
Supplement: Supplementary file 1 [file cancers-14-03472-s001.zip › cancers-1806422-supplementary.pdf]

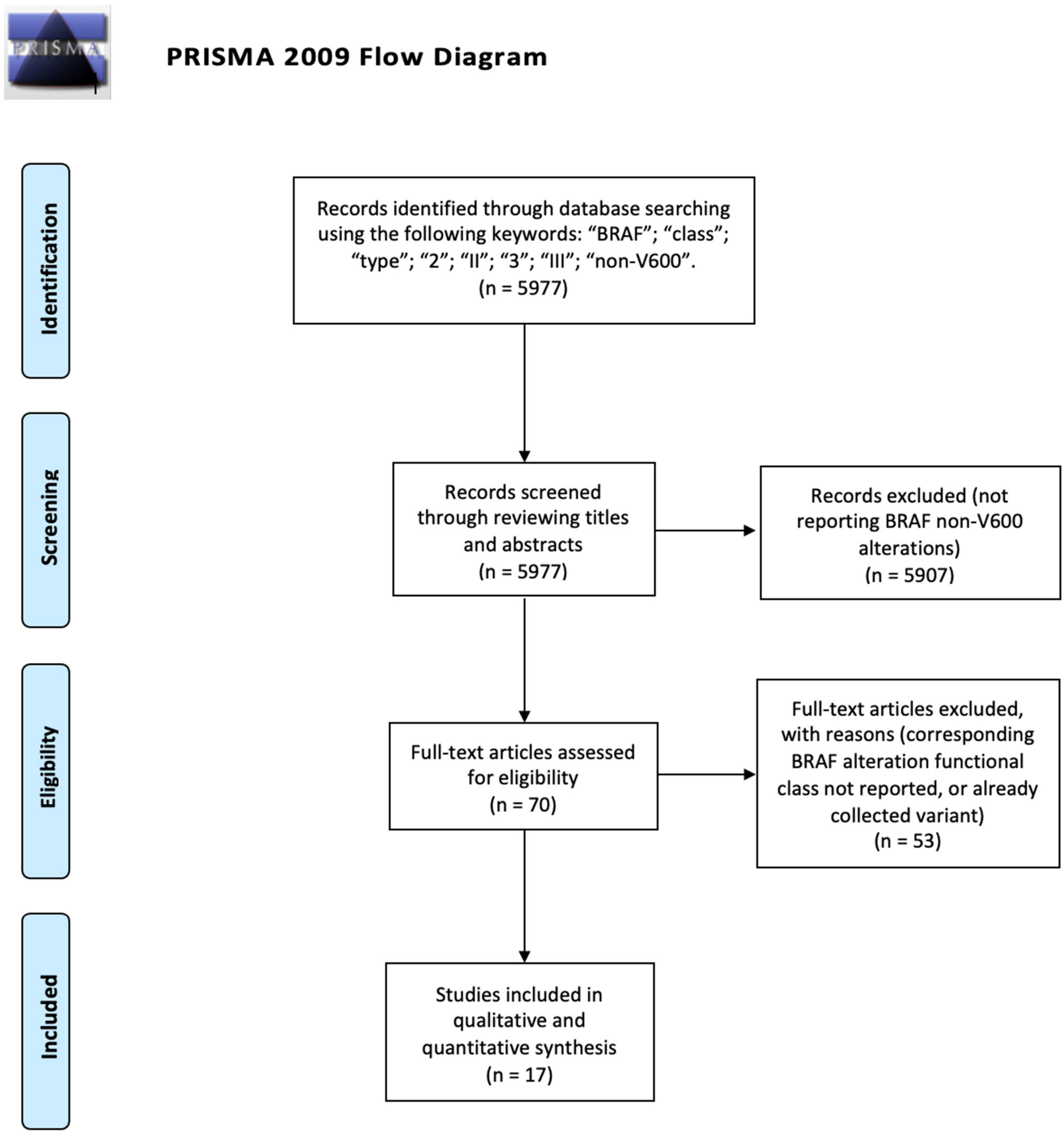

Figure S1. Preferred Reported Items for Systematic Reviews and Meta-Analysis (PRISMA) flowchart of literature research adopted to conduct the systematic review.

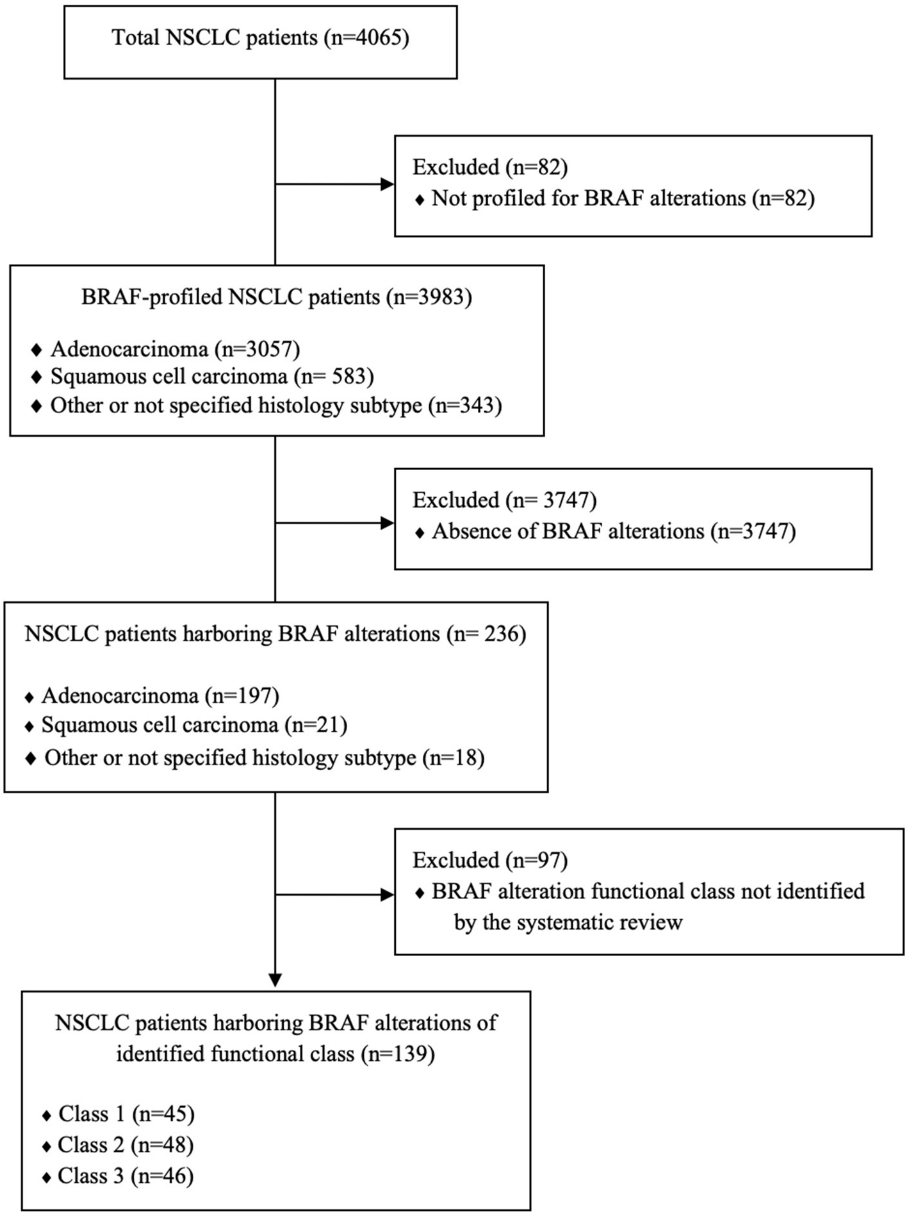

Figure S2. Consort Diagram summarizing the selection of NSCLC patients harboring BRAF alterations of defined functional class in the cBioPortal.

Table S1. List of BRAF alterations (protein change) and corresponding functional class detected through the systematic review of the literature.

Class 2	Class 3
A598V	D287H
A712T	D287Y
A727V	D594A
E451Q	D594E
E586K	D594G
Fusions	D594H
G464A	D594N
G464E	D594V
G464R	D594Y
G464V	F247L
G469A	F595L
G469L	G466A
G469R	G466E
G469S	G466R
G469V	G466V
I463S	G469E
K499E	G569R
K601E	G596A
K601N	G596C
K601Q	G596D
K601T	G596R
L485_P490delinsY	K483E
L485F	K483M
L505F	N581I
L505H	N581K
L525R	N581S
L597Q	N581T
L597R	N581Y
L597S	Q524L
L597V	R558Q
N486_P490del	S467A
Q257R	S467E
R462I	S467L
T599_V600TinsT	T599A
T599dup	V459L
T599I	
T599R	
V471F	
V487_P492delinsA	
V600_K601delinsE	
V600_K601delinsEN	
V600_S605delinsEISRWR	

Table S2. Comparative frequency of concurrent gene alterations in most commonly altered and key genes among NSCLC patients harboring BRAF class 1, class 2, and class 3 alterations. RAS includes alterations of HRAS, NRAS, and KRAS genes. DDR includes alterations in BRCA1, BRCA2, PARP1, PARP2, ERCC1, MLH1, MSH3, MSH2, MSH6, PMS2, ATM, CHEK2, SLFN11, ATR, CHEK1, WEE1, FANCD2, RAD51, AURKA, PALB2, and TP53 genes. NSCLC: non-small cell lung cancer; DDR: DNA damage response and repair genes.

Gene	Class 1, %	Class 2, %	Class 3, %	*p value* for group differences	*p value* class 1 vs class 2	*p value* class 1 vs class 3	*p value* class 2 vs class 3	Enriched class
**SETD2**	**44**	**2**	**7**	**0,000000043**	**0,000017**	**0,00018**	0,60918	**1**
**STK11**	**0**	**21**	**33**	**0,000225**	**0,00732**	**0,00028**	0,2899	**3**
**POM121L12**	**0**	**0**	**35**	**0,001**	**-**	**0,037**	**0,037**	**3**
**TERT**	**0**	**26**	**17**	**0,002**	**0,0026**	**0,0210**	0,482	**2**
**MUC16**	**13**	**33**	**70**	**0,002**	0,3539	**0,0084**	0,1060	**3**
**OVCH1**	**7**	**44**	**5**	**0,003**	0,084	1	**0,040**	**2**
**ZFHX4**	**13**	**28**	**65**	**0,004**	0,560	**0,020**	0,098	**3**
**ITGA4**	**0**	**0**	**30**	**0,004**	-	0,074	0,074	**3**
**KEAP1**	**2**	**22**	**26**	**0,005**	**0,0226**	**0,0094**	0,8069	**3**
**RAS**	**0**	**21**	**13**	**0,006**	**0,0098**	0,0743	0,437	**2**
**XIRP2**	**7**	**17**	**50**	**0,008**	0,733	0,054	0,137	**3**
**CREBBP**	**2**	**12**	**0**	**0,012**	0,28	0,99	0,12	**2**
**KRAS**	**0**	**15**	**4**	**0,013**	0,069	0,484	0,364	2
**CTNNA2**	**0**	**39**	**15**	**0,015**	0,065	0,387	0,387	**2**
**GRIA3**	**0**	**22**	**0**	**0,015**	0,18	-	0,18	**2**
**TFAP2D**	**7**	**0**	**30**	**0,016**	0,93	0,40	0,11	**3**
**GRM8**	**7**	**0**	**30**	**0,016**	0,93	0,40	0,11	**3**
**JAKMIP2**	**0**	**6**	**30**	**0,017**	1	0,18	0,26	**3**
**KLHL41**	**20**	**0**	**0**	**0,018**	0,28	0,28	-	**1**
**UMODL1**	**20**	**0**	**0**	**0,018**	0,28	0,28	-	**1**
**DNAH5**	**0**	**33**	**40**	**0,021**	0,087	0,052	0,929	**3**
**ABCA13**	**7**	**17**	**45**	**0,021**	0,73	0,11	0,25	**3**
**TP53**	**38**	**58**	**65**	**0,024**	0,152	**0,048**	0,635	**3**
**PKHD1L1**	**7**	**6**	**35**	**0,025**	1	0,23	0,20	**3**
**CNTNAP2**	**7**	**11**	**40**	**0,026**	1	0,2	0,2	**3**
**SDHA**	**0**	**15**	**9**	**0,028**	0,059	0,261	0,520	**2**
**PTPRD**	**4**	**19**	**24**	**0,03**	0,128	0,056	0,759	**3**
**FSTL5**	**0**	**17**	**35**	**0,031**	0,587	0,098	0,587	**3**
**FYB1**	**0**	**17**	**35**	**0,031**	0,587	0,098	0,587	**3**
**CSMD1**	**0**	**33**	**35**	**0,033**	0,098	0,098	1	**3**
**FAT1**	**2**	**20**	**15**	**0,033**	0,063	0,138	0,783	**2**
**FLG2**	**0**	**33**	**15**	**0,038**	0,13	0,68	0,68	**2**
**HRNR**	**0**	**33**	**15**	**0,038**	0,13	0,68	0,68	**2**
**AHNAK2**	**20**	**6**	**40**	**0,039**	0,74	0,74	0,10	**3**
**CPLANE1**	**0**	**22**	**35**	**0,04**	0,316	0,098	0,611	**3**
**FBXL7**	**0**	**22**	**35**	**0,04**	0,316	0,098	0,611	**3**
**FGFR1/2/3/4**	**0**	**13**	**13**	**0,042**	0,11	0,11	1	**2,3**
**RYR2**	**13**	**39**	**55**	**0,042**	0,42	0,09	0,50	**3**
**SI**	**0**	**11**	**30**	**0,042**	0,61	0,18	0,61	**3**
**IL1RAPL1**	**0**	**11**	**30**	**0,042**	0,61	0,18	0,61	**3**
**CDH10**	**7**	**44**	**40**	**0,043**	0,13	0,13	1	**2**
**ADAMTS16**	**0**	**33**	**30**	**0,045**	0,13	0,13	1	**2**

**ARAP2**	**0**	**17**	**0**	**0,045**	0,39	-	0,39	**2**
**ALK**	**0**	**0**	**7**	**0,047**	1	0,47	0,47	3
**DDR genes**	**49**	**69**	**72**	**0,049**	0,17	0,13	0,93	**3**
**COL11A1**	**7**	**17**	**40**	**0,05**	0,73	0,20	0,44	**3**
BRCA1/2	2	17	9	0,051	-	-	-	2
MUC2	0	33	20	0,051	-	-	-	2
HCN1	0	33	20	0,051	-	-	-	2
CDH6	0	33	20	0,051	-	-	-	2
TTN	20	50	60	0,056	-	-	-	3
MET	0	4	11	0,057	-	-	-	3
PTPRT	2	17	11	0,057	-	-	-	2
ROS1	0	11	4	0,06	-	-	-	2
MMP16	7	11	35	0,062	-	-	-	3
C6	0	28	30	0,064	-	-	-	3
HOXB6	13	0	0	0,072	-	-	-	1
CNTNAP1	13	0	0	0,072	-	-	-	1
HLA-DQB1	13	0	0	0,072	-	-	-	1
CCL5	13	0	0	0,072	-	-	-	1
PEX12	13	0	0	0,072	-	-	-	1
HEATR9	13	0	0	0,072	-	-	-	1
SNORD7	13	0	0	0,072	-	-	-	1
AP2B1	13	0	0	0,072	-	-	-	1
TAF15	13	0	0	0,072	-	-	-	1
RGPD4	13	0	25	0,076	-	-	-	3
RSF1	20	0	5	0,083	-	-	-	1
ERICH3	0	28	25	0,086	-	-	-	2
SLC1A3	0	28	25	0,086	-	-	-	2
NTRK1/2/3	4	19	11	0,087	-	-	-	2
SMARCA4	4	4	15	0,089	-	-	-	3
COL3A1	0	11	25	0,092	-	-	-	3
SDK1	0	11	25	0,092	-	-	-	3
NF1	2	15	13	0,096	-	-	-	2
TRIO	0	28	20	0,096	-	-	-	2
CDH12	7	17	35	0,107	-	-	-	3
EGFR	7	21	11	0,111	-	-	-	2
LRP1B	13	26	45	0,118	-	-	-	3
LRFN5	0	17	25	0,12	-	-	-	3
C7	0	17	25	0,12	-	-	-	3
WDR70	0	17	25	0,12	-	-	-	3
LRP2	0	17	25	0,12	-	-	-	3
NUP155	0	17	25	0,12	-	-	-	3
GDNF	0	17	25	0,12	-	-	-	3
MUC17	13	17	40	0,122	-	-	-	3
ZNF804A	13	17	40	0,122	-	-	-	3
DNAH3	20	17	0	0,123	-	-	-	1
PXDNL	13	28	45	0,125	-	-	-	3
LAMB4	0	22	10	0,13	-	-	-	2
AFF2	7	11	30	0,137	-	-	-	3
FSIP2	7	11	30	0,137	-	-	-	3
MROH2B	7	28	35	0,144	-	-	-	3
HMGCS1	0	22	20	0,153	-	-	-	2

PKHD1	0	22	20	0,153	-	-	-	2
DNAJC21	0	22	20	0,153	-	-	-	2
UGT3A1	0	22	20	0,153	-	-	-	2
NRXN1	0	22	15	0,164	-	-	-	2
AGXT2	0	22	15	0,164	-	-	-	2
PRKAA1	0	22	15	0,164	-	-	-	2
TMEM267	0	22	15	0,164	-	-	-	2
C5ORF34	0	22	15	0,164	-	-	-	2
CARD6	0	22	15	0,164	-	-	-	2
MTAP	0	17	5	0,169	-	-	-	2
TTC33	0	17	5	0,169	-	-	-	2
COL7A1	7	33	20	0,17	-	-	-	2
MUC5B	7	28	10	0,174	-	-	-	2
APC	2	12	9	0,18	-	-	-	2
EGFLAM	0	17	20	0,194	-	-	-	3
SKP2	0	17	20	0,194	-	-	-	3
DAB2	0	17	20	0,194	-	-	-	3
TTC23L	0	17	20	0,194	-	-	-	3
PTGER4	0	17	20	0,194	-	-	-	3
SYNE2	20	11	35	0,205	-	-	-	3
NEB	7	28	30	0,215	-	-	-	3
ANK2	7	28	30	0,215	-	-	-	3
RET	0	4	7	0,239	-	-	-	3
HLA-DQA1	13	6	0	0,24	-	-	-	1
HCG23	13	6	0	0,24	-	-	-	1
GAS2L2	13	6	0	0,24	-	-	-	1
HLA-DRB1	13	6	0	0,24	-	-	-	1
HLA-DRA	13	6	0	0,24	-	-	-	1
HLA-DRB6	13	6	0	0,24	-	-	-	1
HLA-DRB5	13	6	0	0,24	-	-	-	1
BTNL2	13	6	0	0,24	-	-	-	1
MARK1	13	0	5	0,253	-	-	-	1
MMP28	13	0	5	0,253	-	-	-	1
TRIB1	13	0	5	0,253	-	-	-	1
PLPPR4	0	17	15	0,26	-	-	-	2
SNORD72	0	17	15	0,26	-	-	-	2
MAP1A	13	11	0	0,262	-	-	-	1
CDKN2A	7	12	17	0,295	-	-	-	3
NOTCH1/2/3/4	13	26	24	0,3	-	-	-	2
PIK3CA	7	15	7	0,306	-	-	-	2
FAT3	20	39	20	0,336	-	-	-	2
PDGFRA	2	4	9	0,354	-	-	-	3
USH2A	27	44	50	0,364	-	-	-	3
ASPM	20	28	10	0,373	-	-	-	1
CACNA1C	20	17	5	0,374	-	-	-	1
MAGEA4	13	6	20	0,422	-	-	-	3
NIPBL	13	17	30	0,422	-	-	-	3
FBN2	13	22	30	0,506	-	-	-	3
VCAN	13	22	30	0,506	-	-	-	3
RYR3	13	22	30	0,506	-	-	-	3
UBAP2L	13	22	10	0,561	-	-	-	2

ADGRV1	20	22	10	0,566	-	-	-	2
MCCC2	13	6	5	0,604	-	-	-	1
MUC6	13	6	5	0,604	-	-	-	1
TSBP1	13	6	5	0,604	-	-	-	1
SCAF4	13	6	5	0,604	-	-	-	1
KMT2C	9	11	15	0,629	-	-	-	3
PSKH2	9	11	15	0,629	-	-	-	3
PCLO	20	11	11	0,653	-	-	-	1
FLNC	13	17	25	0,654	-	-	-	3
DOCK11	13	11	5	0,675	-	-	-	1
DMD	20	33	30	0,683	-	-	-	2
SMAD4	4	6	9	0,71	-	-	-	3
TRPM6	13	6	10	0,744	-	-	-	1
MAP2K1	4	2	2	0,753	-	-	-	1
CSMD3	40	50	50	0,806	-	-	-	2,3
CTNNB1	7	6	4	0,874	-	-	-	1
MYC	7	9	9	0,925	-	-	-	2,3
NAV3	13	17	15	0,965	-	-	-	2

Table S3. Descriptions of detected BRAF structural variants in cBioPortal.

Event type	N of patients with the event	Event info	Significance
Fusion	2	PJA2-BRAF fusion	Likely oncogenic, likely gain-of-function
Fusion	1	AGK-BRAF fusion	Oncogenic, gain-of-function
Fusion	1	BRAF-SND1 fusion	Oncogenic, gain-of-function
Fusion	1	AGAP3-BRAF fusion	Likely oncogenic, likely gain-of-function
Fusion	1	BRAF-TRIM24 fusion	Likely oncogenic, gain-of-function
Fusion	1	BRAF-ZC3HAV1 fusion	Likely oncogenic, likely gain-of-function
Fusion	1*	BRAF-LRRC16A fusion	Likely oncogenic, likely gain-of-function
Deletion	1*	BRAF deletion, intragenic	Unknow oncogenic and biological effects
Deletion	1	BRAF deletion, intragenic	Unknow oncogenic and biological effects

* These events occurred in the same patients.
